# A Chemical Mutagenesis Approach to Insert Post-translational Modifications in Aggregation-Prone Proteins

**DOI:** 10.1021/acschemneuro.2c00077

**Published:** 2022-05-24

**Authors:** Ying Ge, Athina Masoura, Jingzhou Yang, Francesco A. Aprile

**Affiliations:** † Department of Chemistry, Molecular Sciences Research Hub, Imperial College London, London W12 0BZ, United Kingdom; ‡ Institute of Chemical Biology, Molecular Sciences Research Hub, Imperial College London, London W12 0BZ, United Kingdom

**Keywords:** post-translational modification, chemical mutagenesis, amyloid-β, Alzheimer’s disease

## Abstract

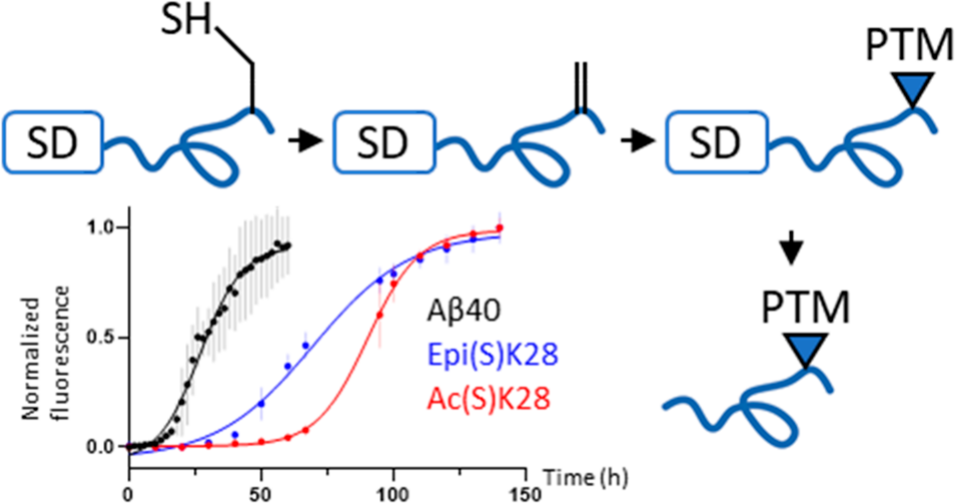

Neurodegenerative diseases are a class of disorders linked to the formation in the nervous system of fibrillar protein aggregates called amyloids. This aggregation process is affected by a variety of post-translational modifications, whose specific mechanisms are not fully understood yet. Emerging chemical mutagenesis technology is currently striving to address the challenge of introducing protein post-translational modifications, while maintaining the stability and solubility of the proteins during the modification reaction. Several amyloidogenic proteins are highly aggregation-prone, and current modification procedures can lead to unexpected precipitation of these proteins, affecting their yield and downstream characterization. Here, we present a method for maintaining amyloidogenic protein solubility during chemical mutagenesis. As proof-of-principle, we applied our method to mimic the phosphorylation of serine-26 and the acetylation of lysine-28 of the 40-residue long variant of amyloid-β peptide, whose aggregation is linked to Alzheimer’s disease.

## Introduction

Dementia is an umbrella term that refers to several pathologies characterized by progressive and irreversible damage to the nervous system. They are a major cause of morbidity and mortality across the globe, with over 78 million people estimated to be affected by 2030 worldwide.^1^ Alzheimer’s disease (AD), the most common form of dementia, is distinguished by disease hallmarks including amyloid-β (Aβ) plaques and tau-containing neurofibrillary tangles.^2,3^ Aβ is an aggregation-prone peptide produced from aberrant cleavage of the amyloid precursor protein (APP), an integral membrane protein abundant at the synapses, by the sequential proteolytic cleavage by β- and γ-secretases.^4^ Genetic mutations of APP and of the secretase genes promoting this proteolytic pathway are linked to familial AD.^5−7^ Furthermore, most cases of AD are sporadic, and post-translational modifications (PTMs) can be induced by environmental factors such as inflammation.^8^ PTMs, including truncation, serine phosphorylation, lysine acetylation, methionine oxidation, and polyglutamylation, are found in Aβ aggregates, with reported effects such as increasing or reducing the rate of aggregation and plaque formation.^2,8^ However, detailed biomolecular and mechanistic studies of each PTM are hindered by the lack of site-specific tools to introduce them. To date, PTMs in Aβ have been generated by solid-state peptide synthesis,^9−11^ enzymatic reaction,^12,13^ and non-site-specific chemical modification.^14^ However, these methods have their drawbacks. Solid-state synthesis is costly. Enzymatic reactions are limited in scope, while chemical modifications tend to react with multiple residues of the same type. In contrast, site-specificity and product versatility can be combined using a dehydroalanine (Dha)-based method of introducing PTM mimetics developed by Davis and co-workers.^15,16^ Briefly, cysteine is converted to Dha via an alkylation–elimination reaction with a dibromide compound, allowing subsequent Michael addition with a variety of thiol-containing compounds. As cysteine can be introduced via molecular cloning techniques, installation of PTMs can be carried out in a site-specific yet versatile manner.

This method has been applied to histone H3,^15,16^ as well as to a single-domain antibody and the amyloidogenic protein tau.^17,18^ However, additional challenges are faced in the modification of Aβ due to its hydrophobic and aggregation-prone nature. Procedures required for the chemical reactions, such as heating and shaking, could lead to protein precipitation and aggregation, thus reducing the yield and limiting downstream application. Here, we establish a facile, high-yield protocol of introducing PTM mimetics in Aβ, while maintaining the peptide solubility, by utilizing a solubility-enhancing domain.^19^ As a proof of concept, we installed mimetics of two PTMs: serine (S) phosphorylation and lysine (K) acetylation. In particular, we modified K16, S26, and K28. K28 has been reported to form a salt bridge with D23 in Aβ40^11,20^ and with the carboxyl group of A42 in Aβ42.^21^ K16 acetylated and K16/K28 double acetylated Aβ42 form amorphous aggregates instead of fibrils, while exhibiting greater cytotoxicity.^10,14^ In contrast, K28 acetylation does not disrupt the fibrillization of Aβ42.^10^ In Aβ40, AcK28 was shown to slow down aggregation kinetics.^22^ S26 phosphorylation in Aβ40 resulted in an alternative pS26–K28 salt bridge. This modification appears to promote the accumulation of cytotoxic oligomeric species while reducing the formation of fibrils, leading to a reduction of fluorescence intensity in thioflavin T (ThT) and Congo red assays.^11,13^ By successfully introducing mimetics of these PTMs, we overcame the unique difficulties of carrying out chemical biology on a peptide with a high propensity to aggregate. We also demonstrate that the aggregation behaviors of these peptides agree with previous reports.^11,13,22^ This method can be readily adapted to introduce other types of PTMs in Aβ and enable a wide range of biophysical studies.

## Results and Discussion

### Experimental Strategy

**Figure 1:**
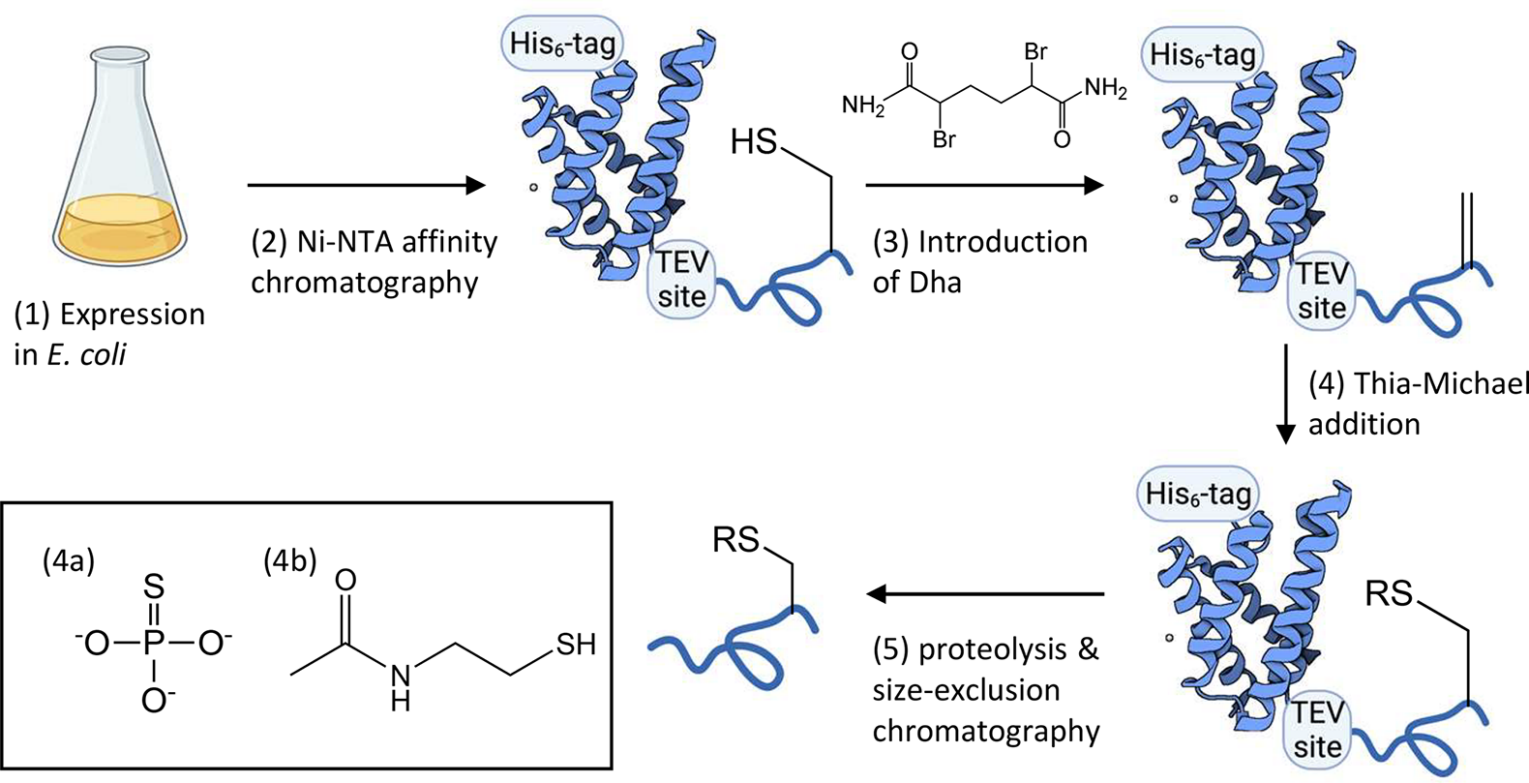
Experimental strategy. (1) SD–Aβ40 with a single cysteine was expressed as a His_6_-tagged fusion protein in *E. coli* BL21(DE3) cells. (2) The fusion protein was purified from the cell lysate by Ni-NTA affinity chromatography. (3) Purified fusion protein was reacted with DBHDA to convert the cysteine residue into a Dha. (4) Dha was then converted into a phosphocysteine or acetyllysine mimic by thia-Michael addition with sodium thiophosphate (4a, R = PO_3_) or *N*-acetylcysteamine (4b, R = C_4_H_8_NO), respectively. (5) The SD was removed via proteolysis by the addition of TEV protease, and the digestion mixture was subjected to size-exclusion chromatography to remove the SD and TEV and to obtain monomeric Aβ. Structure of the SD (PDB 4FBS) was rendered as cartoon using BioRender.

Aβ40 and its cysteine variants were expressed in *Escherichia coli* (*E. coli*) as a fusion protein with spider silk protein domain (SD) as previously reported.^19^ Purification and modification were carried out on the fusion protein (Figure 1), as we theorize that enhanced solubility afforded by the SD would allow multiple steps of chemical modification to be carried out on Aβ. First, SD–Aβ40 (or Aβ40 variant) was purified under reducing conditions using affinity chromatography, via its N-terminal His_6_ tag. As there is no cysteine present in Aβ40 or SD natively, the introduced cysteine could be site-specifically converted to Dha by reaction with 2,5-dibromohexanediamide (DBHDA). Dha was then reacted with sodium thiophosphate, *N*-acetylcysteamine, or cysteamine to introduce PTM mimetics of phosphoserine, acetyllysine, and lysine, respectively, in a thia-Michael addition reaction. Finally, the addition of tobacco etch virus (TEV) protease separates the SD from Aβ, and size-exclusion chromatography (SEC) enables the removal of SD to obtain modified Aβ. To show the general applicability of this strategy, we produced Aβ40 variants carrying modifications at K16, S26, and K28.

### Introduction of Dha and PTM Mimetics to SD–Aβ40

**Figure 2:**
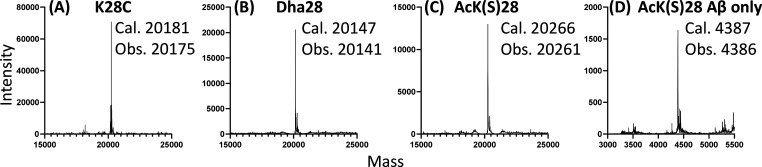
Deconvoluted mass spectra of SD–Aβ40-K28C (A) before modification, (B) after introduction of the Dha intermediate, (C) after introduction of the acetyllysine mimetic, and (D) as a monomeric peptide. Cal., calculated mass; Obs., observed mass.

SD–Aβ40-S26C, SD–Aβ40-K28C, and SD–Aβ40-K16C were purified as fusion proteins by Ni-NTA affinity chromatography. Mass spectrometry (MS) confirmed the serine or lysine to cysteine mutations, as the observed mass matches that of the expected fusion protein minus an N-terminal methionine, which is thought to have been lost during purification or MS analysis (Figures 2 and S1–S14). In SD–Aβ40-K16C and SD–Aβ40-K28C, additional peaks with an approximately +76 mass shift were observed (Figures 2B, S5, and S11), which may be a β-mercaptoethanol (BME) adduct resulting from the purification process. These mass shifts disappear after reaction with DBHDA to introduce Dha, indicating that they do not interfere with the alkylation–elimination process. Instead, a mass shift of −34 Da, compared to the starting material without BME-adduct, was observed (Figures 2B,F, S2, and S6) following incubation of the protein with DBHDA at 37 °C for 3 h, indicating the conversion of the cysteine into Dha (SD–Aβ40-Dha26 or SD–Aβ40-Dha28).

Following the introduction of the acetyllysine mimic, a mass shift of +120 Da from Dha was observed (Figures 2C and S7). The resulting products contain a sulfur in place of Cγ in (acetyl)lysine and are hereafter referred to as “Ac(S)K”.

Similar mass shifts were observed in SD–Aβ40-K28C (Figures S5–S8) and SD–Aβ40-K16C (Figures S11–S14), suggesting that the efficiency of the protocol is unaffected by the location of the target modification or its surrounding residues.

For SD–Aβ40-Dha26, phosphoserine mimic phosphocysteine was introduced (product denoted SD–Aβ40-pC26) after reaction with sodium thiophosphate, in accordance with a mass shift of +80 Da compared to SD–Aβ40-S26C (Figures S3 and S15).

### Purification of Monomeric Aβ40 Variants

**Figure 3:**
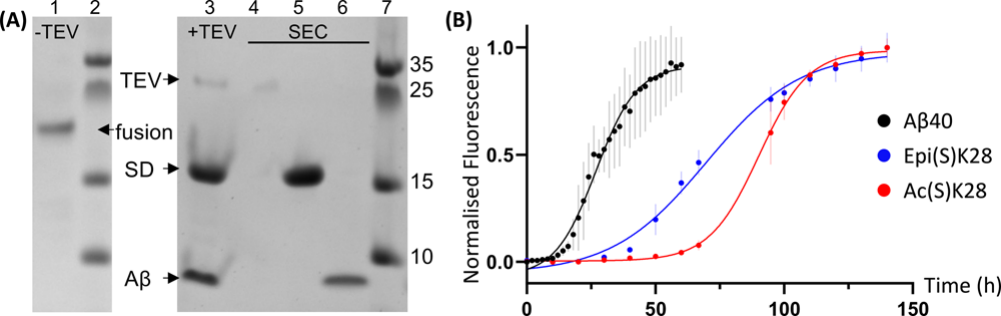
(A) Representative SDS-PAGE showing the Aβ40 (or PTM variants) as a fusion protein (lane 1), after reaction with TEV (lane 3), and after purification by size exclusion chromatography (lanes 4–6). (B) ThT aggregation assay of 5 μM recombinant Aβ40 (black), Aβ40-Ac(S)K28 (red), and Aβ40-Epi(S)K28 (blue). Fluorescence of 20 μM ThT in buffer alone was recorded in parallel and subtracted as baseline. Error bars show standard deviation (*N* = 5). Data were normalized and fitted to a sigmoidal curve in Prism.

**Figure 4:**
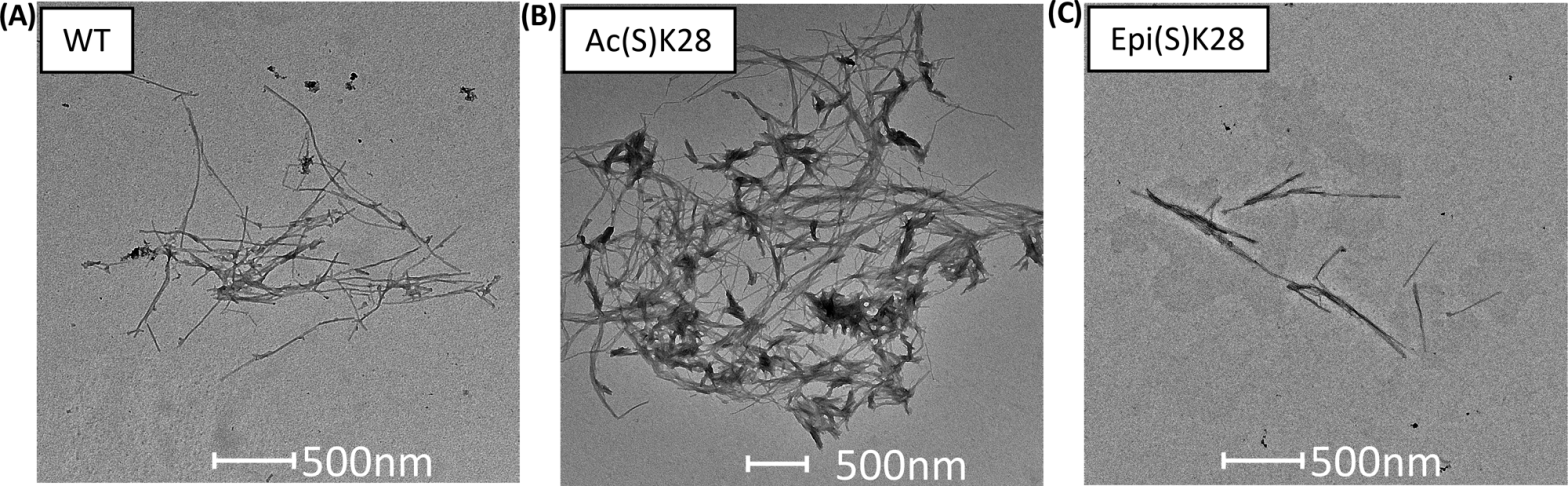
TEM images showing fibrils of (A) Aβ40, (B) Aβ40-Ac(S)K28, and (C) Aβ40-Epi(S)K28 taken during the plateau phase of aggregation.

To produce authentic (i.e., with no overhanging initial methionine) Aβ40 peptide without the SD, the fusion protein was incubated with TEV protease to remove the N-terminal silk domain (Figures 1 and 3A). The resulting peptide begins with an aspartic acid, as do naturally occurring Aβ peptides. The proteolyzed sample was subjected to SEC, and fractions containing purified Aβ peptides were collected for subsequent characterization (Figures 3 and 4). MS analysis showed Aβ40 peptide at the expected mass with high homogeneity (Figures 2D, S4, S8, S10, and S14), confirming that the modifications remain stable after removal of the silk domain by TEV protease cleavage and the SEC process. From the SEC chromatograms (Figure S16), it appears that during removal of the guanidinium the Aβ40 variants run at comparable retention volumes, suggesting they have the same conformation.

### Comparison of the Aggregation Behaviors of Aβ40 and PTM Variants

ThT assays were carried out to compare the aggregation of Aβ40 and the modified variants to determine whether our modifications have consistent effects with the PTMs of which they are mimetics. Compared to wild-type Aβ40, Ac(S)K28 resulted in slower aggregation (Figure 3B). The data show effects on both the lag phase and growth phase, indicating that multiple nucleation mechanisms are affected. Transmission electron microscopy (TEM) on samples taken at the plateau of the aggregation showed long, thin fibrils for Aβ40 (Figure 4A). Shorter, heavily clustered fibrils were observed for Aβ40-Ac(S)K28 (Figure 4B), suggesting that K28 acetylation affects the kinetics as well as the morphology of aggregates. Due to the planar conformation of Dha, it is likely that the final products are an epimeric mixture with l- and d-conformations at position 28. To account for the effect of epimerization, as well as that of having a sulfur atom in place of Cγ, we reacted Aβ40-Dha28 with cysteamine and obtained a variant that we named Aβ40-Epi(S)K28 (Figures S9 and S10). This variant aggregated more slowly than wild-type Aβ40 but more rapidly than the corresponding acetylated variant (Figure 3B). This observation indicates that epimerization and acetylation both affect the rate of aggregation. However, so long as an epimeric control is included, the effect resulting from PTM can be isolated. TEM images showed thin fibrils that are closer in morphology to those of wild-type (Figure 4C). These results suggest that epimerization affects the kinetics of aggregation but not the structure of the fibrils.

Aβ40-pC26 showed a slow, close-to-linear increase in ThT fluorescence (Figure S15E), similar to solid-phase synthesized Aβ40-pS26.^11,13^ A lack of fibrillar aggregates observable by TEM in Aβ40-pC26 samples after incubation (Figure S15F) confirms the disruption to fibrillization caused by phosphorylation of residue 26.

The current study is a proof of concept of a strategy to post-translationally modify in a site-specific manner aggregation-prone proteins. It is particularly suited for intrinsically disordered proteins, given their solvent accessibility. Several works have demonstrated the suitability of these PTM mimetics as research tools. Histone H3 containing pC generated via the Dha intermediate was shown to respond to pS-specific antibodies and to be a substrate of phosphatases.^16^ Installing pC at S356 of tau results in the inhibition of tubulin polymerization, an effect also observed when S356 is phosphorylated by other methods.^18^ Our results further demonstrated that PTM mimetics introduced via the Dha intermediate can be used to study the effect of the corresponding PTMs on protein aggregation. Although the effect of epimerization is not negligible, it can be isolated from that of the PTM mimetic as long as a control is included.

## Methods

### Cloning, Protein Expression and Purification of the Silk Domain–Aβ40 Fusion Proteins

The mutations S26C, K16C, and K28C were introduced to the SD–Aβ40 fusion protein^19^ via site-directed mutagenesis using a QuikChange Lightning Kit (Agilent) following a modified protocol.^23^ The successful introduction of mutations was confirmed by sequencing (Genewiz). Chemically competent *E. coli* BL21(DE3) cells were transformed with the wild-type or mutated plasmids and used to overexpress proteins according to an established protocol.^19^ Briefly, cells were cultured in LB–kanamycin medium at 37 °C, 120 rpm, until OD_600nm_ reached 0.8–0.9, when the temperature was lowered to 20 °C and expression was induced by the addition of 0.1 mM isopropyl β-d-1-thiogalactopyranoside (IPTG). After incubation overnight, cells were harvested by centrifugation at 6000 *g* for 15 min, and the pellets were frozen and thawed prior to resuspension in binding buffer (8 M urea, 20 mM Tris-HCl (pH 8.0), 15 mM imidazole) and sonication at 500 W, 20% amplitude, for 10 min (15 s on, 45 s off). Cell lysate was cleared by centrifugation at 38758 *g*, and the supernatant was passed through a syringe filter with a diameter of 0.45 μm. The filtrate was applied to a HisTrap HP column (Cytiva), and the target protein was eluted with a mix of 40% binding buffer and 60% elution buffer (8 M urea, 20 mM Tris-HCl (pH 8.0), 300 mM imidazole). The eluate was collected and dialyzed against 50 mM phosphate buffer (pH 8.0) for 16 h. For cysteine-containing variants, binding and elution buffers were supplemented with 3 mM β-mercaptoethanol (BME). Protein concentrations were calculated using *A*_280nm_ values measured on a Nanodrop (Thermo Scientific), and extinction coefficient values obtained from the Expasy ProtParam tool.^24^

### Introduction of Dha

Tris(2-carboxyethyl)phosphine (TCEP, 2 mM) and 400 mol equiv of DBHDA was added to 200 μM SD–Aβ40-K16C, SD–Aβ40-S26C, or SD–Aβ40-K28C, and the reaction was incubated at 37 °C with shaking at 400 rpm for 3 h. After the precipitate was removed by centrifugation, small molecules were removed by passing the sample through a HiTrap desalting column with Sephadex G-25 resin (Cytiva) pre-equilibrated in 0.1 M sodium phosphate buffer (pH 8.0) or by dialysis. For mass spectrometry (MS) analysis (liquid chromatography electrospray, LC-ES), the samples were desalted using Zeba Spin columns (Thermo Scientific) with 7 kDa MWCO.

### Generation of Phosphocysteine

Sodium thiophosphate (NaSPO_3_, 480 mg) was dissolved in 186 μL of H_2_O and 200 μL of 5 M HCl. This stock was added to 100 μM Dha-containing protein (SD–Aβ40-Dha26) at 1:5 volume ratio. Reactions were carried out at 37 °C with shaking at 400 rpm for 6 h in the presence of 1.5 M urea. Excess salt was removed by dialysis in 0.1 M sodium phosphate buffer (pH 8.0) prior to TEV cleavage. For MS analysis, samples were desalted using Zeba Spin columns (Thermo Scientific) with 7 kDa MWCO.

### Generation of Lysine and Acetyllysine Mimics

*N*-Acetylcysteamine (50 μL) or 56 mg of cysteamine was added to 1.2 mL of 100 μM SD–Aβ40-Dha16 or SD–Aβ40-Dha28. Reactions were incubated at room temperature (25 °C) with shaking at 400 rpm for 3 h. Excess salt was removed by dialysis in 0.1 M sodium phosphate buffer (pH 8.0) prior to TEV cleavage. For MS analysis, samples were desalted using Zeba Spin columns (Thermo Scientific) with 7 kDa MWCO.

### Removal of the SD and Purification of the Aβ40 Variants

SD–Aβ40 and variants were dialyzed against 0.1 M phosphate buffer (pH 8.0), and the SD was cleaved from Aβ40 by incubation with TEV protease at 20:1 molar ratio for 1 h at room temperature followed by 23 h at 4 °C. After proteolysis, the sample was dissolved in 6 M guanidine-HCl and subjected to size exclusion chromatography on a HiLoad 16/600 Superdex 30 pg column (Cytiva) pre-equilibrated in 20 mM phosphate buffer (pH 8.0) supplemented with 200 μM EDTA to separate the Aβ40 peptide from TEV and SD. Fractions were analyzed by SDS-PAGE, and only those containing pure Aβ were used for subsequent applications. Concentration is calculated from the UV absorption reading on an ÄKTA Pure protein purification system operated with the Unicorn 7 software. For MS analysis, the samples were buffer exchanged into H_2_O using an Amicon concentrator with 3 kDa MWCO (Millipore).

### ThT Aggregation Assay

Aβ40 and PTM variants were mixed and incubated with 20 μM ThT and 0.02% sodium azide at 37 °C without shaking in 20 mM sodium phosphate buffer (pH 8.0), 200 μM EDTA, in a black 96-well nonbinding microplate with clear bottom (Greiner no. 655906). Fluorescence was monitored in a CLARIOstar Plus or FLUOstar Omega plate reader with excitation filter of 440 ± 10 nm and emission filter of 480 ± 10 nm.

### Transmission Electron Microscopy

Aβ40 and PTM variants were mixed and incubated with 20 μM ThT and 0.02% sodium azide at 37 °C without shaking in 20 mM sodium phosphate buffer (pH 8.0), 200 μM EDTA. Samples were collected during the plateau phase of incubation, deposited onto carbon-coated copper mesh grids, and negatively stained with 2% (w/v) uranyl acetate. The samples were then viewed with a FEI Tecnai T12 Spirit 120 kV transmission electron microscope.

## Supporting Information Available

Reaction mechiansm of PTM installation via Dha, yield of modification per milligram of starting material, LC-ES MS and deconvolution of Aβ40-S26C as fusion protein,SD–Aβ40-Dha26, SD–Aβ40-pC26, Aβ40-pC26 after TEV cleavage and SEC, SD–Aβ40-K28C, SD–Aβ40-Dha28, SD–Aβ40-Ac(S)K28, Aβ40-Ac(S)K28 after TEV cleavage and SEC, SD–Aβ40-Epi(S)K28, Aβ40-Epi(S)K28 after TEV cleavage and SEC, SD–Aβ40-K16C, SD–Aβ40-Dha16, SD–Aβ40-Ac(S)K16, and Aβ40-Ac(S)K16 after TEV cleavage, introduction of a phosphoserine mimetic (phosphocysteine) at S26 of Aβ40 and characterisation, and SEC of Aβ40, Aβ40-pC26, Aβ40-Ac(S)K28, and Aβ40-Epi(S)K28 (PDF)

## Abbreviations

Aβ: amyloid-β
AcK: acetyllysine
Ac(S)K: thiol-containing mimic of AcK
AD: Alzheimer’s disease
APP: amyloid precursor protein
BME: β-mercaptoethanol
DBHDA: 2,5-dibromohexanediamide
DTT: dithiothreitol
EDTA: ethylenediaminetetraacetic acid
Epi(S)K: thiol-containing epimeric mimic of lysine
IPTG: isopropyl β-D-1-thiogalactopyranoside
LC-ES: liquid chromatography electrospray
MS: mass spectrometry
pC: phosphocysteine
pS: phosphoserine
PTM: post-translational modification
SD: silk domain
SDS-PAGE: sodium dodecyl sulfate–polyacrylamide gel electrophoresis
SEC: size-exclusion chromatography
TCEP: tris(2-carboxyethyl)phosphine
TEM: transmission electron microscopy
TEV: tobacco etch virus
ThT: thioflavin T
