# Supplementary material for: A Chemical Mutagenesis Approach to Insert Post-translational Modifications in Aggregation-Prone Proteins
Source: ACS Chem Neurosci. 2022 May 24;13(12):1714–8. doi: 10.1021/acschemneuro.2c00077 (PMC9204764; doi:10.1021/acschemneuro.2c00077)
Supplement: Supplementary file 1 — cn2c00077_si_001.pdf [file cn2c00077_si_001.pdf]

A Chemical Mutagenesis Approach to Insert Post-Translational Modifications in Aggregation-Prone Proteins

Ying Ge[1], Athina Masoura[1], Jingzhou Yang[1], Francesco A. Aprile[1,2]*

[1]*Department of Chemistry, Molecular Sciences Research Hub, Imperial College London, London W12 0BZ, United Kingdom*

[2]*Institute of Chemical Biology, Molecular Sciences Research Hub, Imperial College London, London W12 0BZ, United Kingdom*

* To whom correspondence should be addressed: f.aprile@imperial.ac.uk

Supplementary information includes:

- Scheme S1
- Table S1
- Figures S1—S16

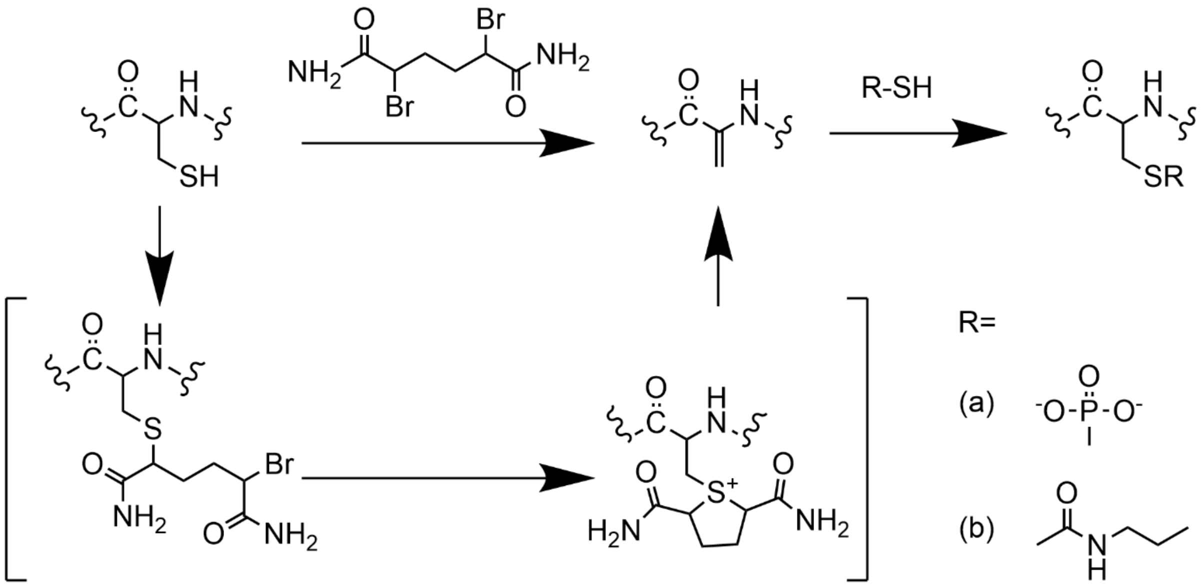

Scheme S1 Reaction mechiansm of PTM installation via Dha.

Table 1 Yield of modification per 1mg of starting material. Yields after each chemical modification step were calculated from concentrations measured on a NanoDrop and sample volumes before and after each reaction. After TEV cleavage and size-exclusion chromatography, yield is determined by peak integration in the Unicorn software.

Reaction	Yield (per 1 mg)
Cys → Dha	0.86 ± 0.12 mg (N = 5)
Dha → Ac(S)K	0.87 ± 0.08 mg (N = 4)
Dha → Epi(S)K	1.0 ± 0.10 mg (N = 2)
TEV + SEC	0.08 ± 0.02 mg (N = 4)<br>0.38 ± 0.09 mg after MW adjustment*
* There is reduction in molecular weight from SD–Aβ40 (20.3 kDa) to monomeric Aβ40 (4.3 kDa) after removal of the SD.	

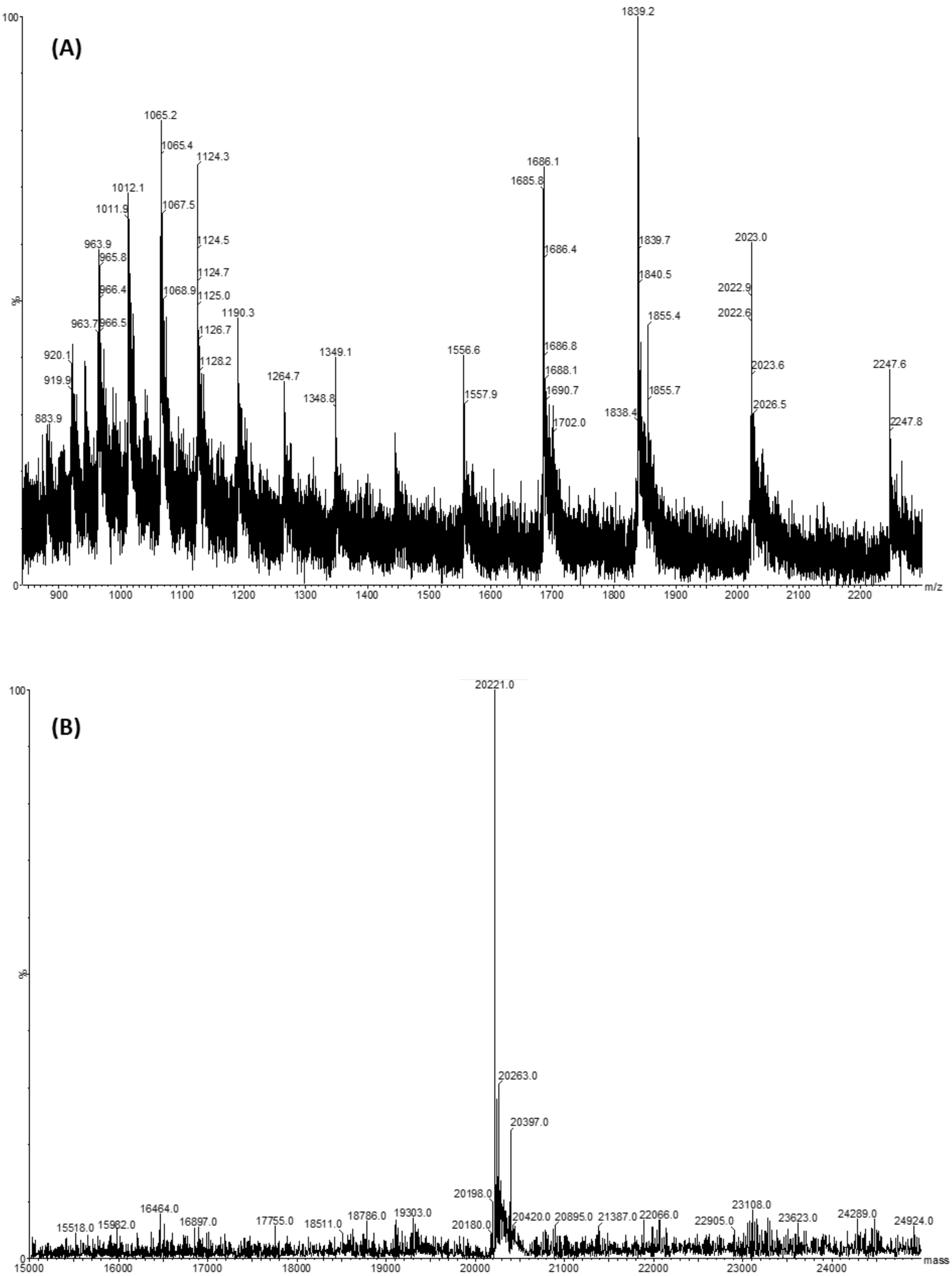

Figure S1. LC-ES mass spectra (A) and deconvolution data (B) of Aβ40-S26C as fusion protein. The calculated mass (minus the N-terminal methionine) is 20222 Da and the observed deconvoluted mass is 20221 Da.

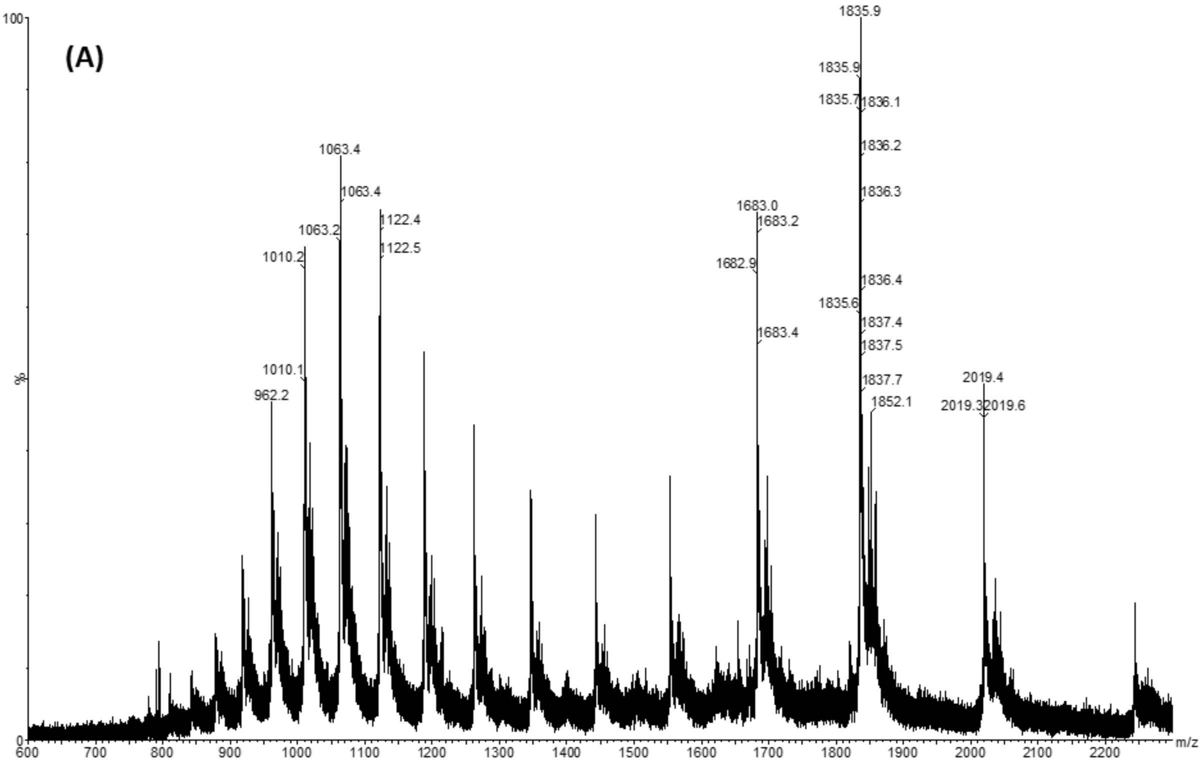

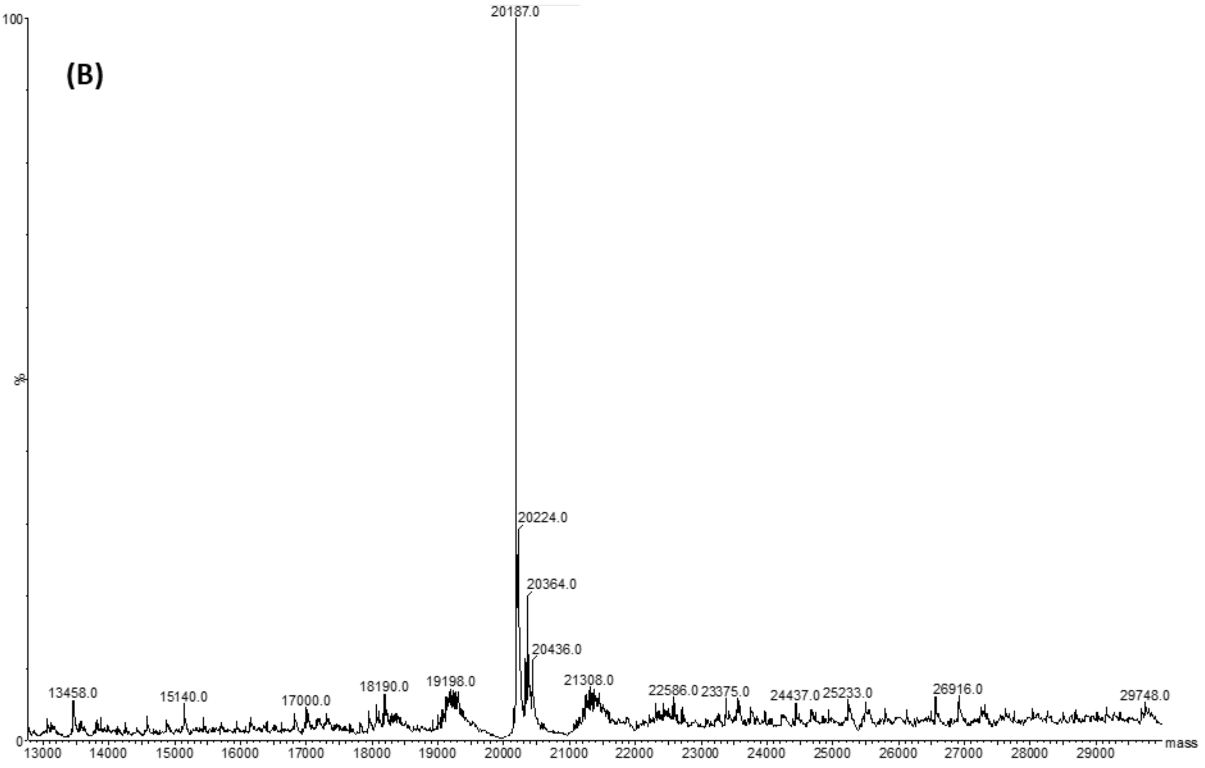

Figure S2. LC-ES mass spectra (A) and deconvolution data (B) of SD–Aβ40-Dha26. The calculated mass (minus the N-terminal methionine) is 20188 Da and the observed deconvoluted mass is 20187 Da. The expected mass shift is –34 Da and the observed mass shift is –34 Da.

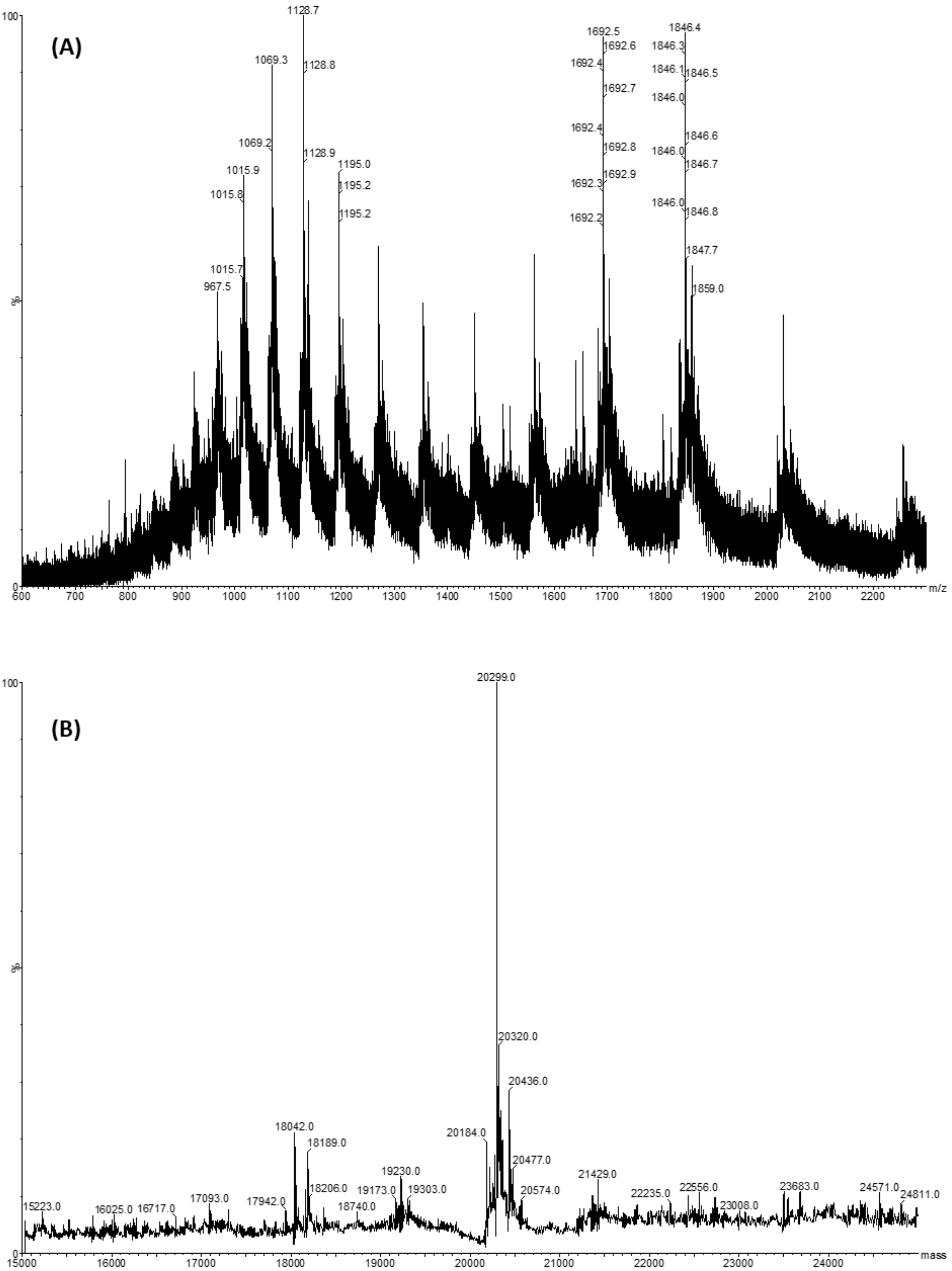

Figure S3. LC-ES mass spectra (A) and deconvolution data (B) of SD–Aβ40-pC26. The calculated mass (minus the N-terminal methionine) is 20302 Da and the observed deconvoluted mass is 20299 Da. The expected mass shift is +80 Da (compared to S26C) and the observed mass shift is +78 Da.

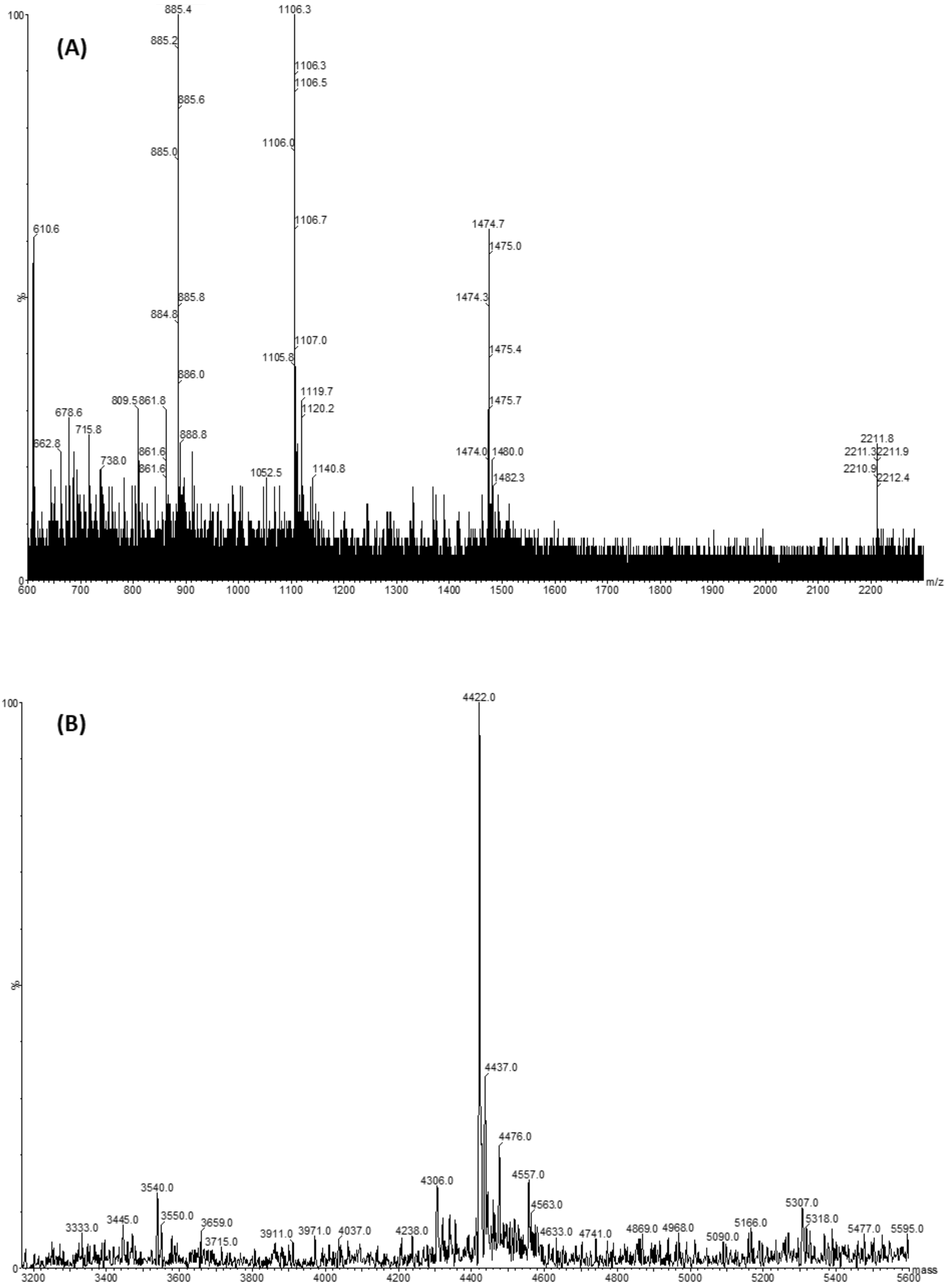

Figure S4. LC-ES mass spectra (A) and deconvolution data (B) of Aβ40-pC26 after TEV cleavage and size-exclusion chromatography. The calculated mass (minus the N-terminal methionine) is 4423 Da and the observed deconvoluted mass is 4422 Da.

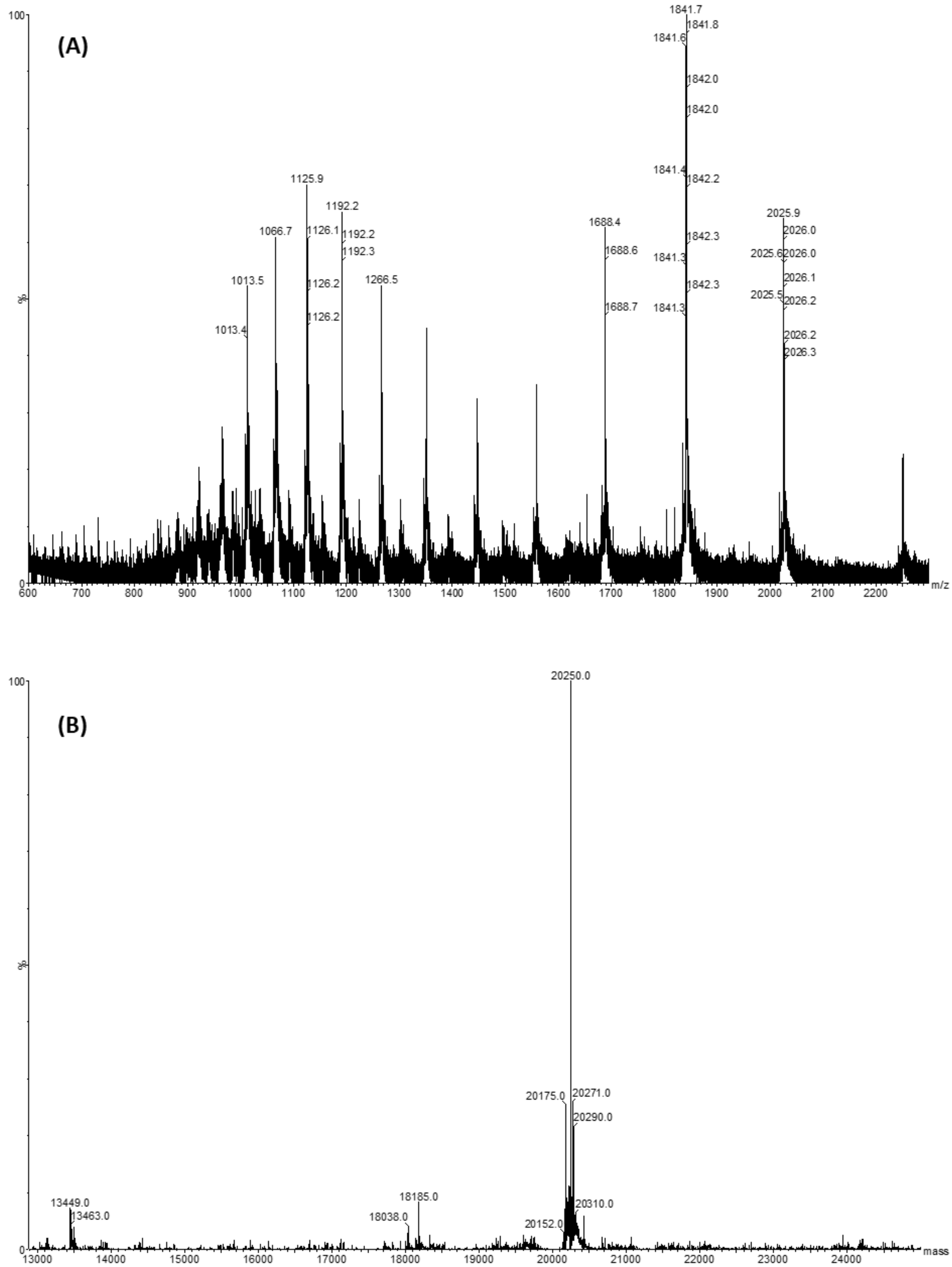

Figure S5. LC-ES mass spectra (A) and deconvolution data (B) of SD–Aβ40-K28C. The calculated mass is 20181 Da and the observed deconvoluted mass is 20175 Da. There is an additional peak at 20250, which may be a BME adduct due to our purification procedure and is absent once the cysteine is converted to Dha.

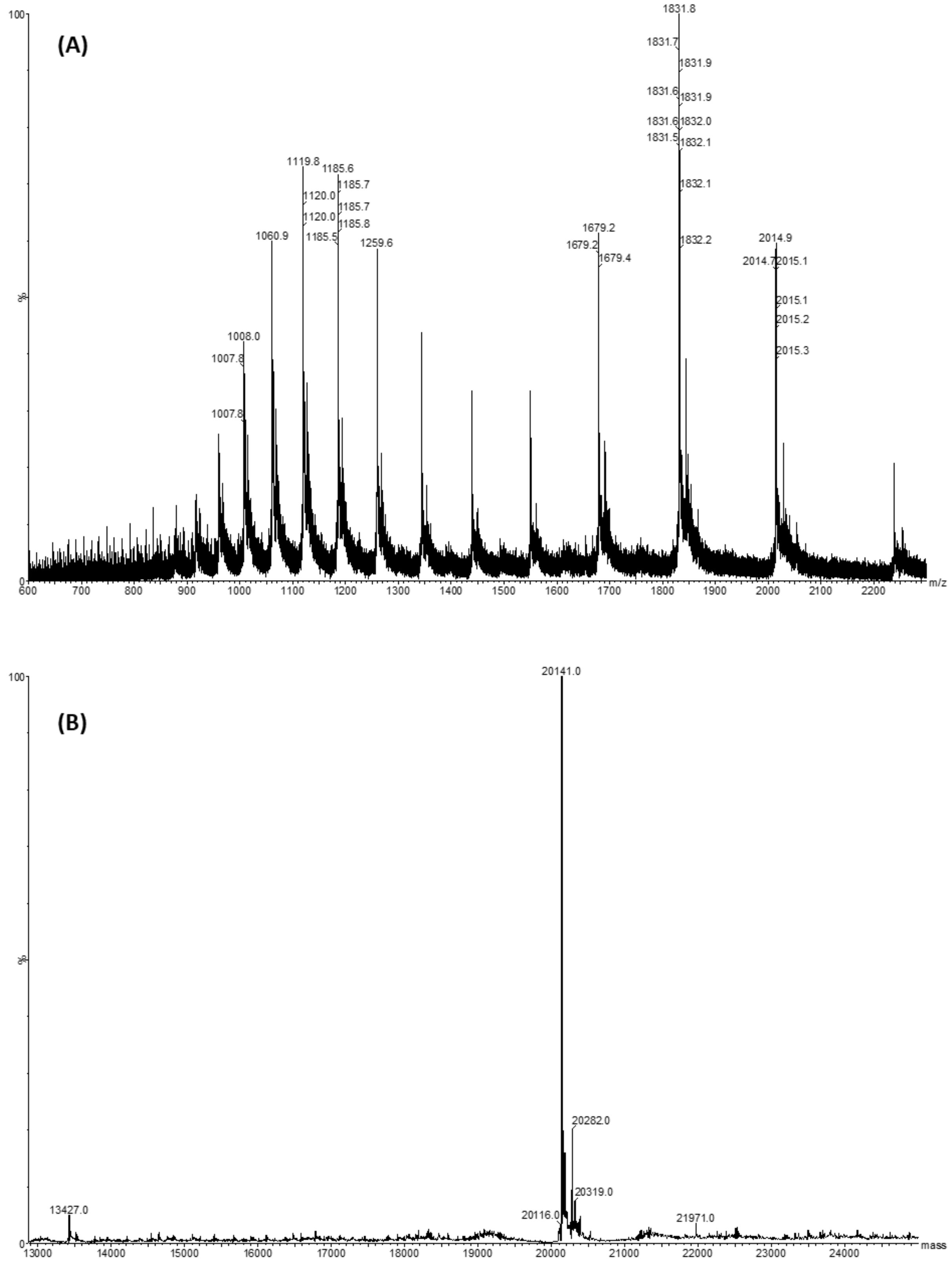

Figure S6. LC-ES mass spectra (A) and deconvolution data (B) of SD–Aβ40-Dha28. The calculated mass (minus the N-terminal methionine) is 20147 Da and the observed deconvoluted mass is 20141 Da. The expected mass shift is –34 Da and the observed mass shift is –34 Da.

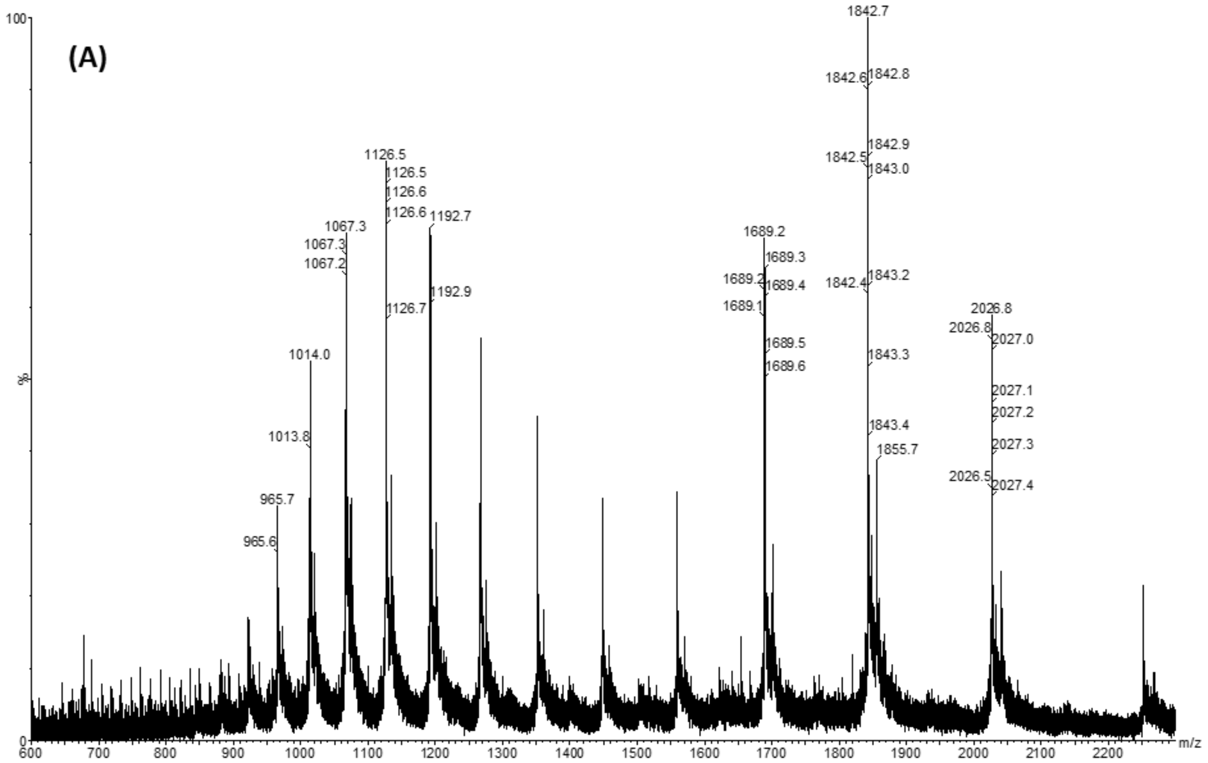

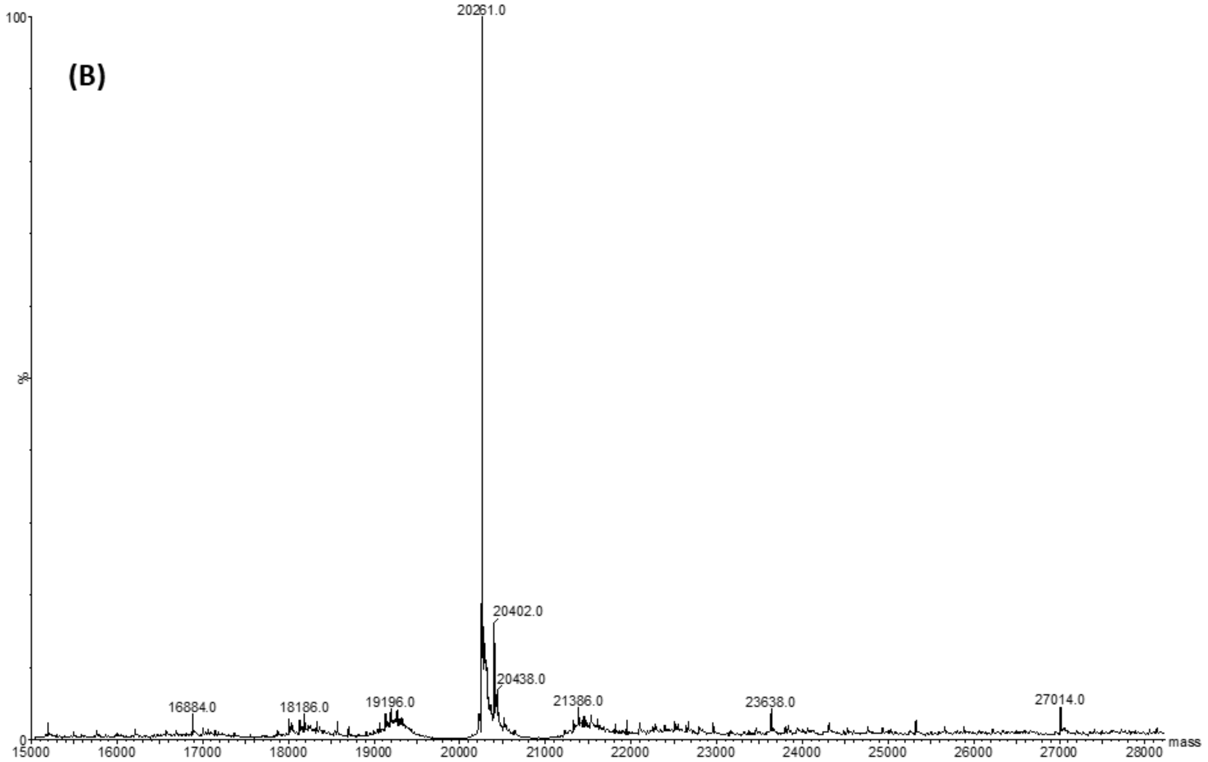

Figure S7. LC-ES mass spectra (A) and deconvolution data (B) of SD–Aβ40-Ac(S)K28. The calculated mass (minus the N-terminal methionine) is 20266 Da and the observed deconvoluted mass is 20261 Da. The expected mass shift is +119 Da from SD–Aβ40-Dha28 and the observed mass shift is +120 Da.

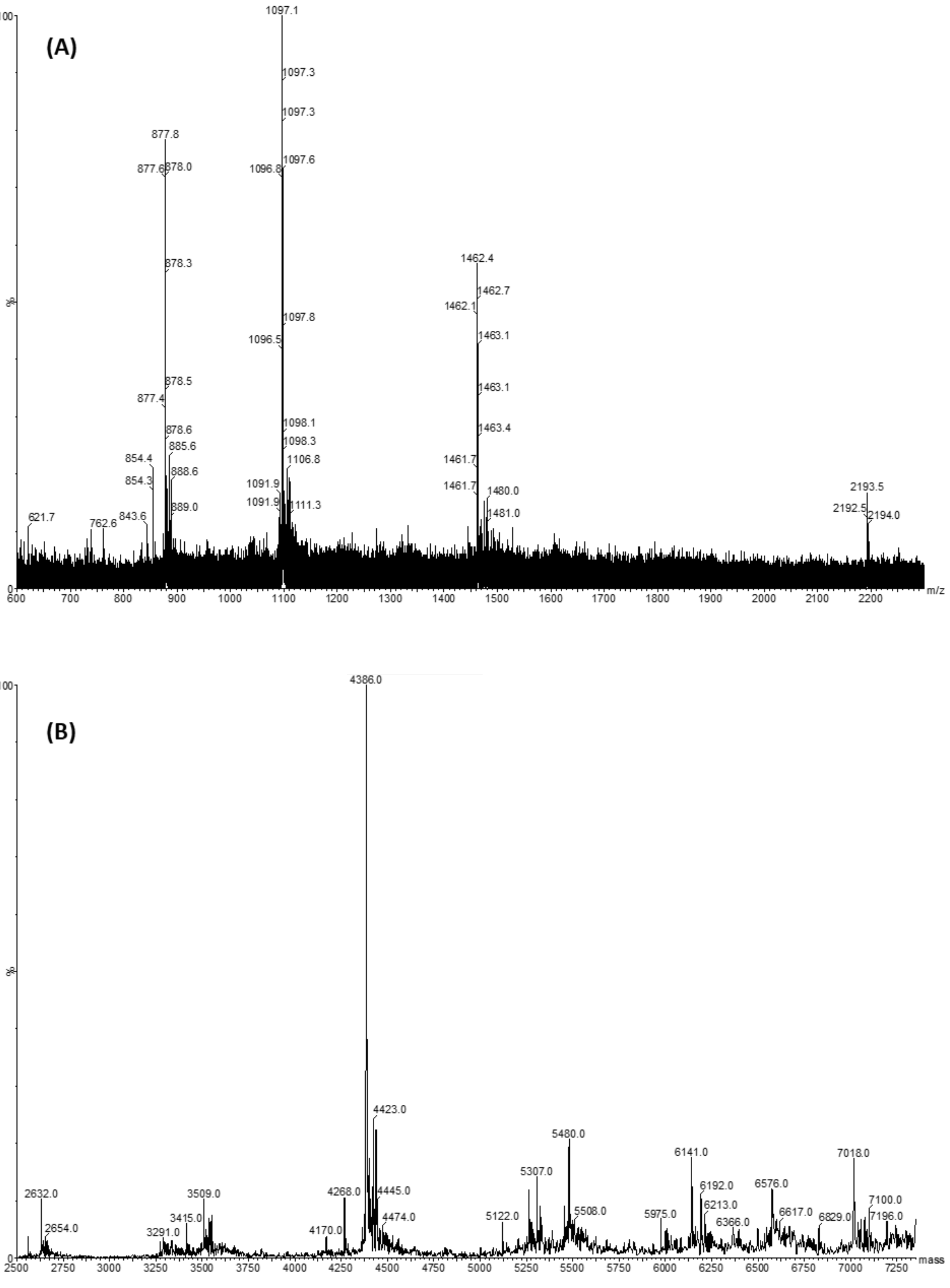

Figure S8. LC-ES mass spectra (A) and deconvolution data (B) of Aβ40-Ac(S)K28 after TEV cleavage and size-exclusion chromatography. The calculated mass (minus the N-terminal methionine) is 4387 Da and the observed deconvoluted mass is 4386 Da.

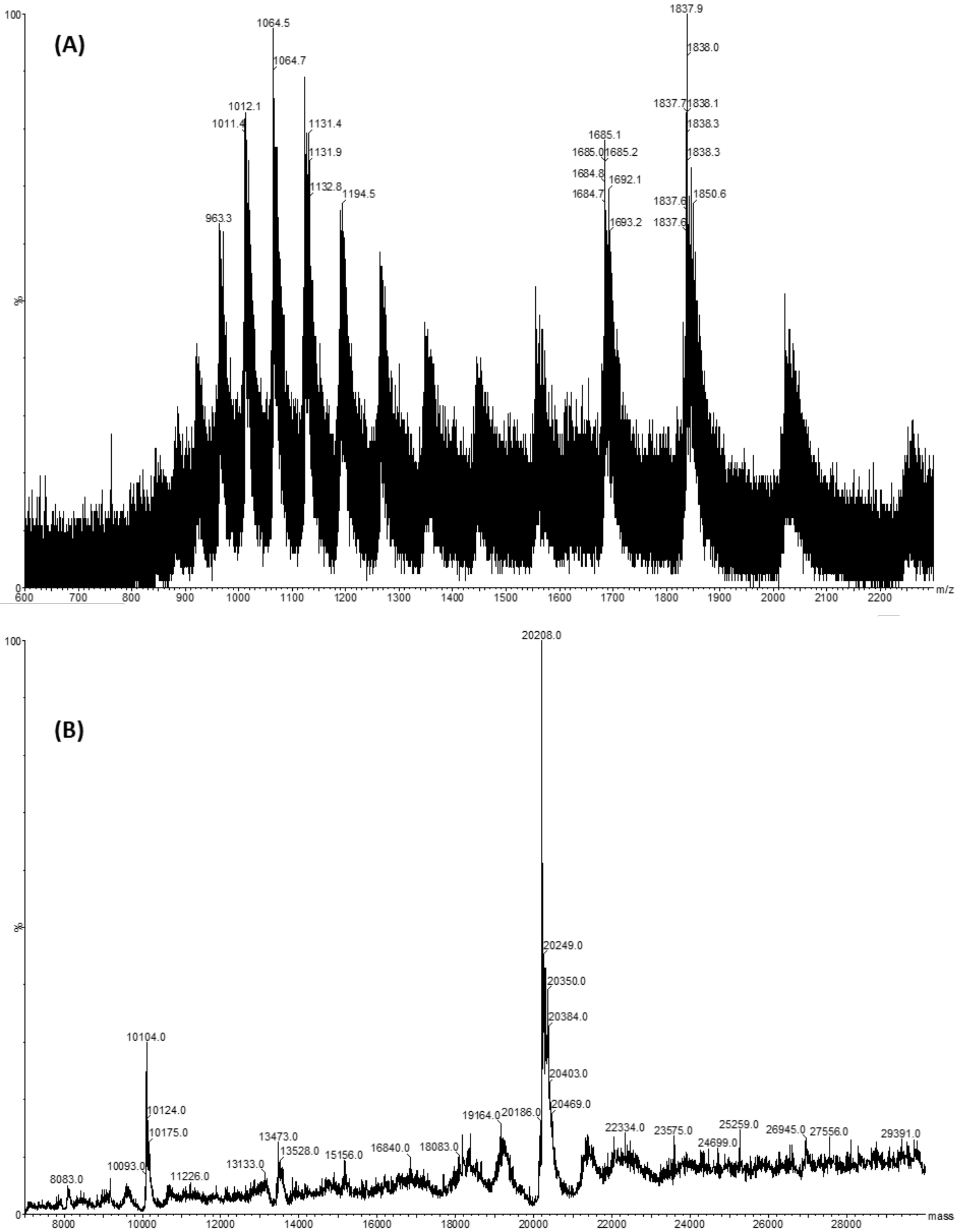

Figure S9. LC-ES mass spectra (A) and deconvolution data (B) of SD–Aβ40-epi(S)K28 (SD–Aβ40-Dha28 reacted with cysteamine). The calculated mass (minus the N-terminal methionine) is 20224 Da and the observed deconvoluted mass is 20206 Da. The expected mass shift is +77 Da from SD–Aβ40-Dha28 and the observed mass shift is +75 Da.

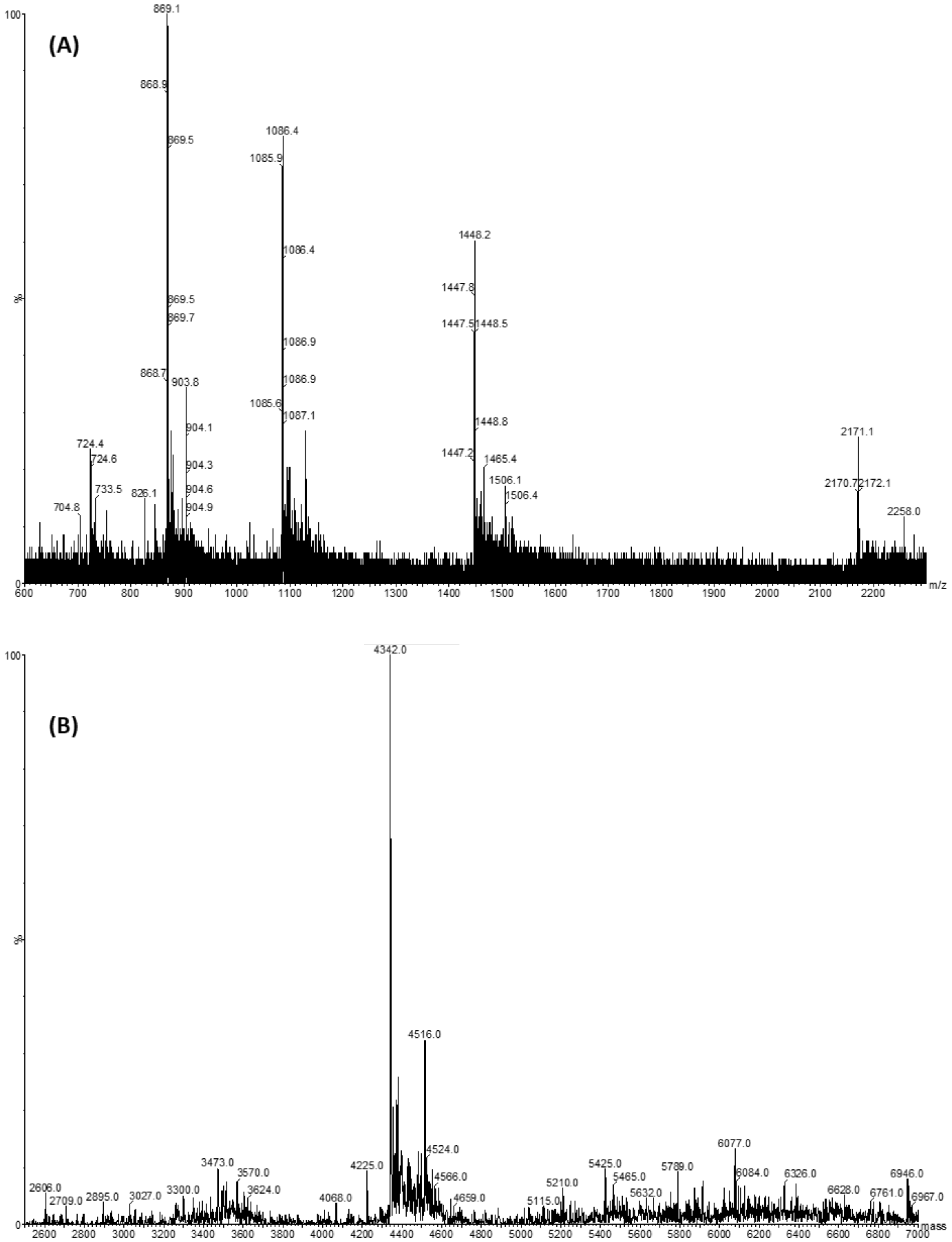

Figure S10. LC-ES mass spectra (A) and deconvolution data (B) of SD–Aβ40-epi(S)K28 after TEV cleavage and size-exclusion chromatography. The calculated mass (minus the N-terminal methionine) is 4345 Da and the observed deconvoluted mass is 4342 Da.

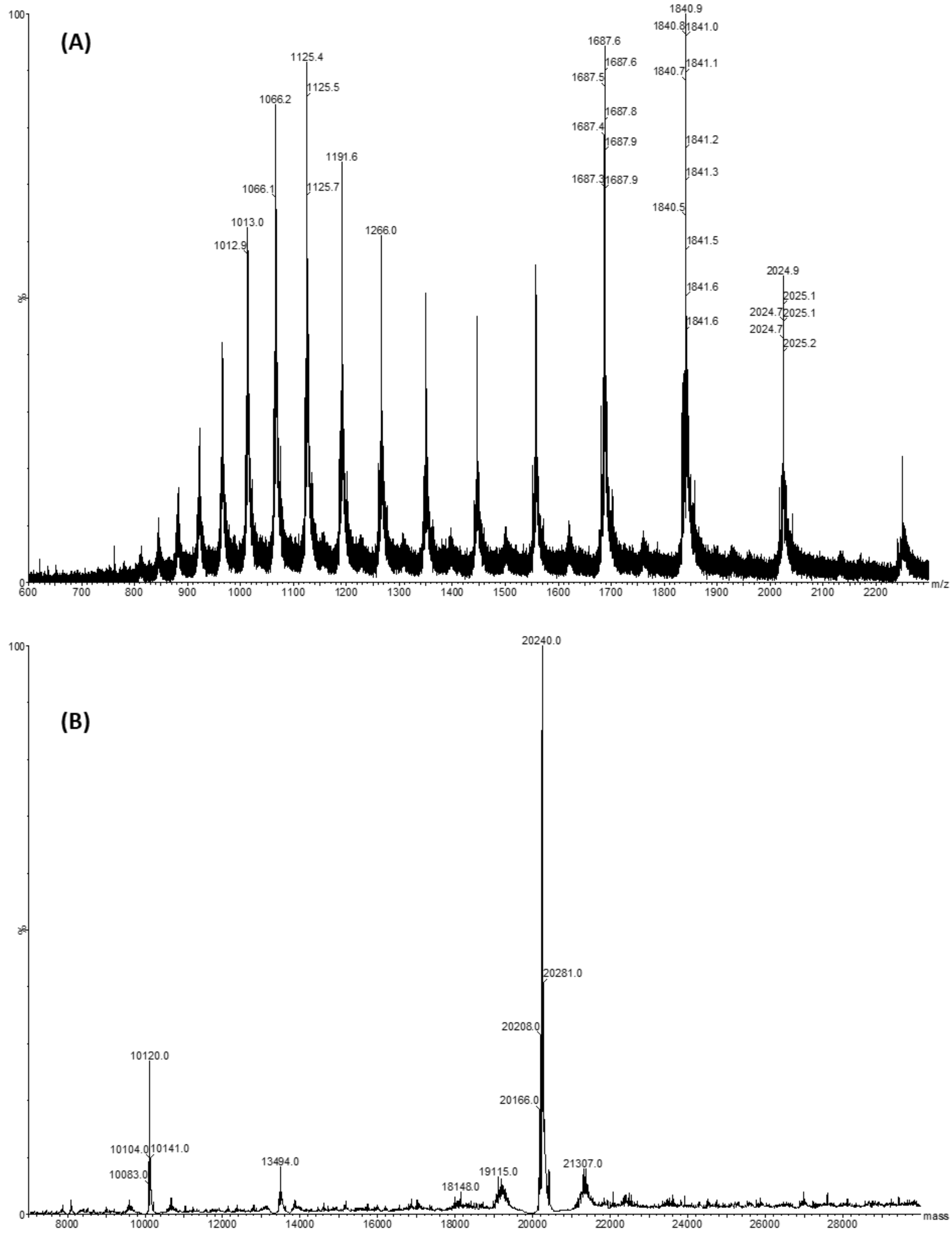

Figure S11. LC-ES mass spectra (A) and deconvolution data (B) of SD–Aβ40-K16C. The calculated mass is 20181 Da (20257 Da with BME adduct) and the observed deconvoluted mass is 20240 Da. The BME adduct becomes absent once the cysteine is converted to Dha.

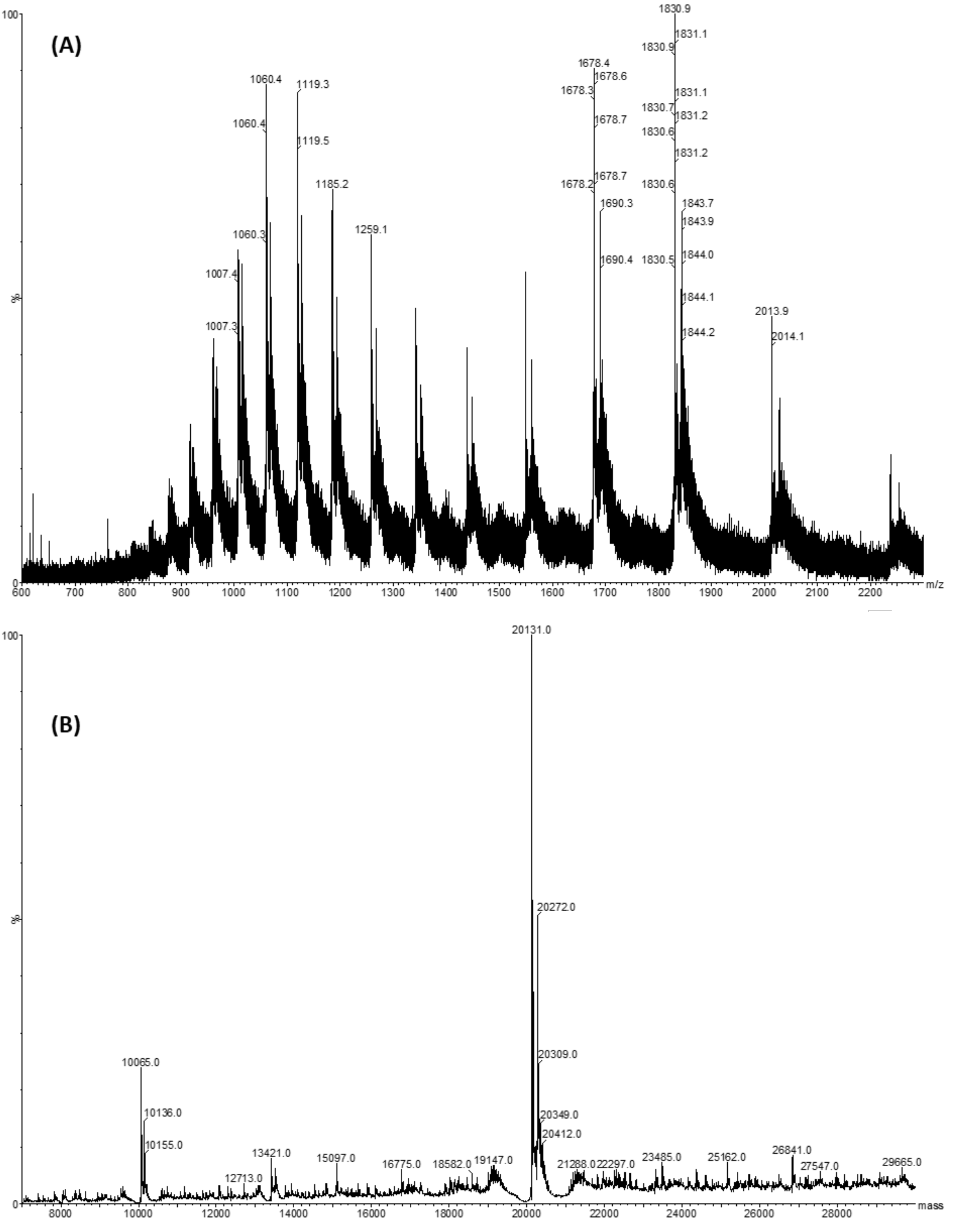

Figure S12. LC-ES mass spectra (A) and deconvolution data (B) of SD–Aβ40-Dha16. The calculated mass (minus the N-terminal methionine) is 20147 Da and the observed deconvoluted mass is 20131 Da. Expected mass shift is –34 Da from SD–Aβ40-K16C and the observed shift is –109 Da due to the BME-adduct (+76 Da) mentioned above in Figure S11.

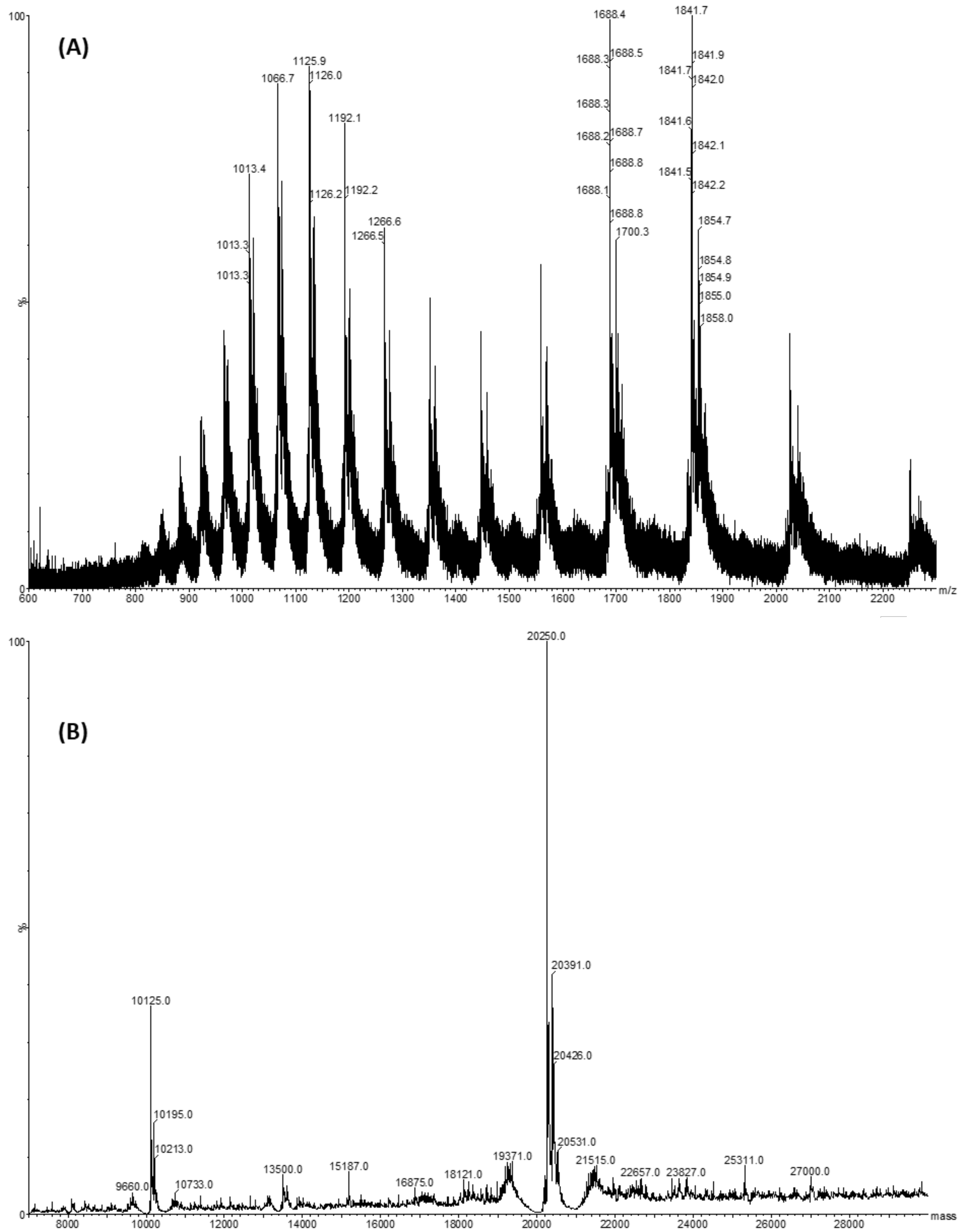

Figure S13. LC-ES mass spectra (A) and deconvolution data (B) of SD–Aβ40-Ac(S)K16. The calculated mass (minus the N-terminal methionine) is 20266 Da and the observed deconvoluted mass is 20250 Da.

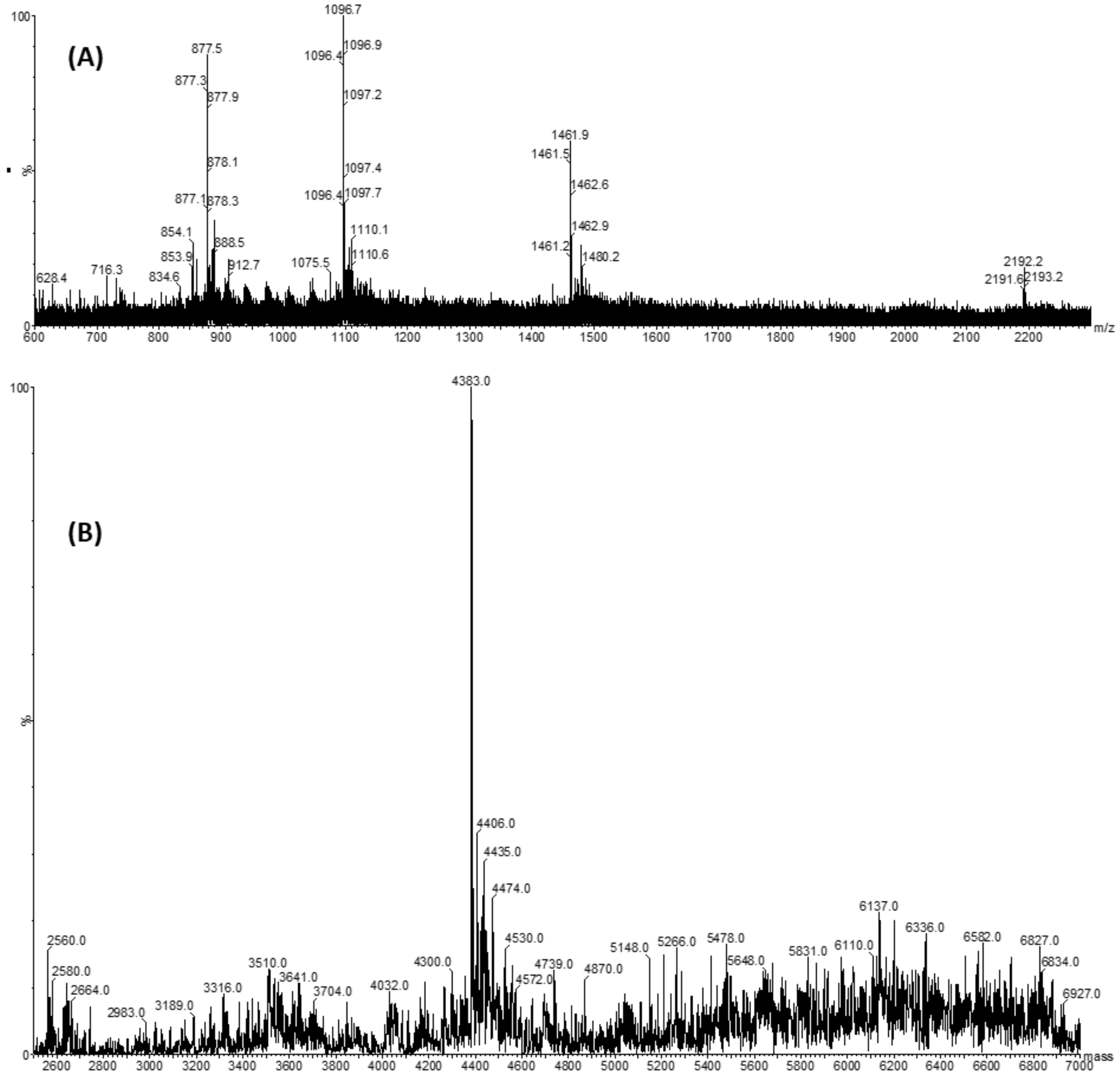

Figure S14. LC-ES mass spectra (A) and deconvolution data (B) of Aβ40-Ac(S)K16 after TEV. The calculated mass (minus the N-terminal methionine) is 4387 Da and the observed deconvoluted mass is 4383 Da.

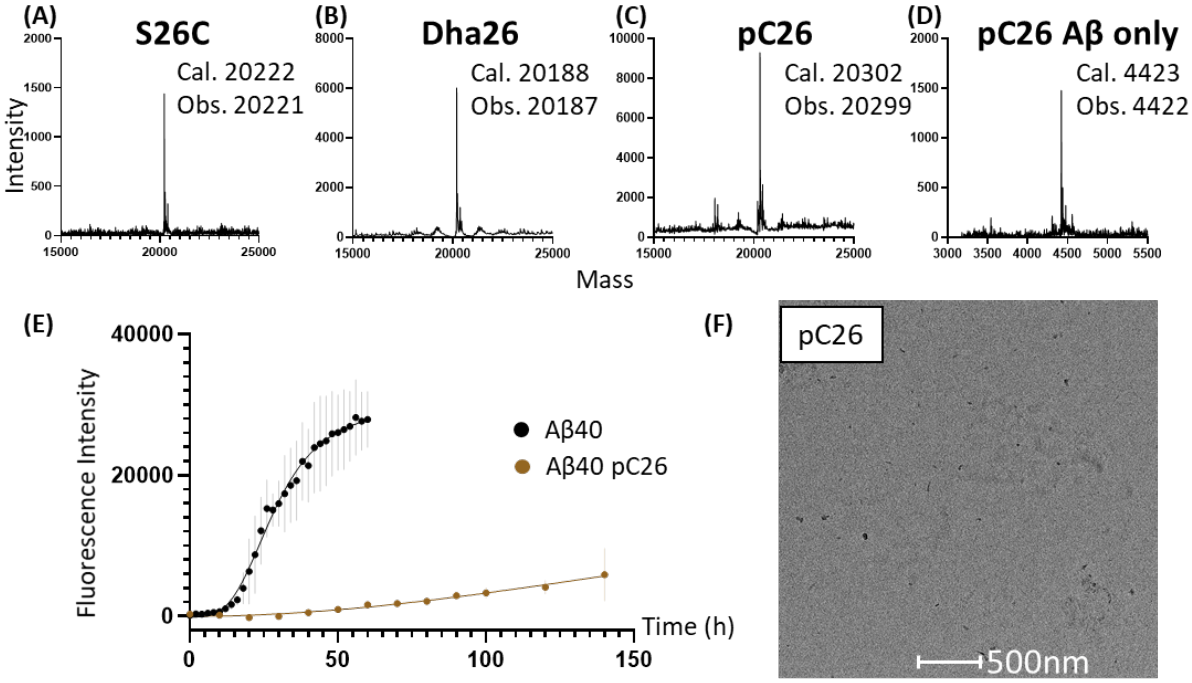

Figure S15. Introduction and characterisation of Aβ40 with a phosphoserine mimetic (phosphocysteine) at S26. (A)-(D) Deconvoluted mass spectra showing the reaction products of each modification step. (E) ThT fluorescence of 5µM wildtype Aβ40 (black) and Aβ40-pC26 (gold) incubated with 20 mM ThT at 37 °C. (F) TEM image of Aβ40-pC26 after 163 h of incubation showing the absence of fibrils.

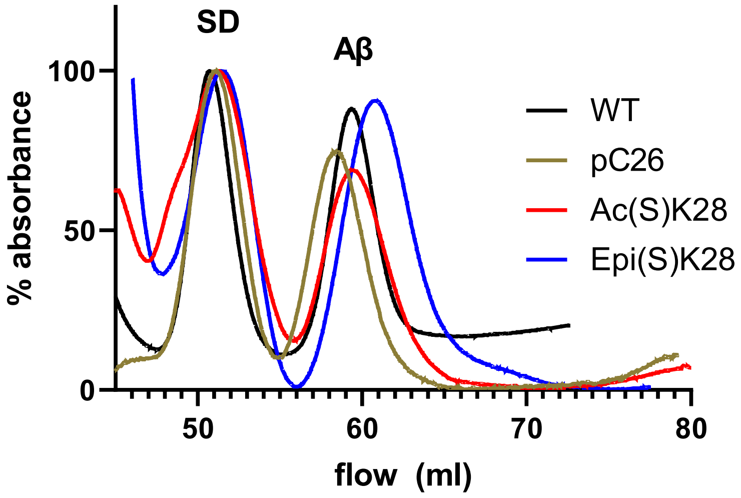

Figure S16. Overlay of size exclusion chromatograms of Aβ40 (black), Aβ40-pC26 (gold) Aβ40-Ac(S)K28 (red) and Aβ40-Epi(S)K28 (blue) showing the separation of SD and monomeric Aβ.
